# Bioinspired Catechol‐Grafting PEDOT Cathode for an All‐Polymer Aqueous Proton Battery with High Voltage and Outstanding Rate Capacity

**DOI:** 10.1002/advs.202103896

**Published:** 2021-12-16

**Authors:** Meihua Zhu, Li Zhao, Qing Ran, Yingchao Zhang, Runchang Peng, Geyu Lu, Xiaoteng Jia, Danming Chao, Caiyun Wang

**Affiliations:** ^1^ College of Chemistry Jilin University Changchun 130012 China; ^2^ State Key Laboratory of Integrated Optoelectronics College of Electronic Science and Engineering Jilin University Changchun 130012 China; ^3^ Key Laboratory of Automobile Materials Ministry of Education School of Materials Science and Engineering Jilin University Changchun 130022 China; ^4^ ARC Centre of Excellence for Electromaterials Science Intelligent Polymer Research Institute AIIM Facility University of Wollongong Wollongong NSW 2522 Australia

**Keywords:** all‐organic batteries, aqueous proton batteries, catechol, poly(3,4‐ethylenedioxythiophene), polymer electrodes

## Abstract

Aqueous all‐polymer proton batteries (APPBs) consisting of redox‐active polymer electrodes are considered safe and clean renewable energy storage sources. However, there remain formidable challenges for APPBs to withstand a high current rate while maximizing high cell output voltage within a narrow electrochemical window of aqueous electrolytes. Here, a capacitive‐type polymer cathode material is designed by grafting poly(3,4‐ethylenedioxythiophene) (PEDOT) with bioinspired redox‐active catechol pendants, which delivers high redox potential (0.60 V vs Ag/AgCl) and remarkable rate capability. The pseudocapacitive‑dominated proton storage mechanism illustrated by the density functional theory (DFT) calculation and electrochemical kinetics analysis is favorable for delivering fast charge/discharge rates. Coupled with a diffusion‐type anthraquinone‐based polymer anode, the APPB offers a high cell voltage of 0.72 V, outstanding rate capability (64.8% capacity retention from 0.5 to 25 A g^−1^), and cycling stability (80% capacity retention over 1000 cycles at 2 A g^−1^), which is superior to the state‐of‐the‐art all‐organic proton batteries. This strategy and insight provided by DFT and ex situ characterizations offer a new perspective on the delicate design of polymer electrode patterns for high‐performance APPBs.

## Graphical abstract

A conductive polymer cathode with high redox potential is realized by grafting catechol to the poly(3,4‐ethylenedioxythiophene) backbone. The all‐polymer aqueous proton cell achieves the high voltage (0.72 V) and current rate (25 A g^−1^) stemming from the significant electron delocalization and pseudocapacitive‑dominated proton storage mechanism. Anew prospect toward high energy/power density for aqueous organic batteries is provided.

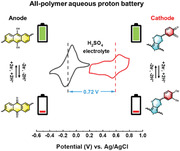

## 1 Introduction

There is an increasing demand to develop affordable, sustainable, and eco‐friendly electrochemical energy storage systems. A rechargeable aqueous battery with various cationic charge carriers is proposed to meet the above requirements, including monovalent ions (H^+^, Li^+^, K^+^, and Na^+^) and multivalent ions (Zn^2+^, Ca^2+^, Mg^2+^, and Al^3+^).^[^ ^1^, ^2^, ^3^, ^4^ ^]^ Although metal‐ion rechargeable batteries possess a high capacity and excellent stability, the ion transport significantly impeded the realization of instantaneous output due to the large ionic radius and strong electrostatic interactions.^[^ ^5^, ^6^ ^]^ Rechargeable aqueous proton batteries based on the proton uptake/removal mechanism are promising to overcome the ion‐diffusion kinetics limit. Protons are allowed to jump rapidly along with the hydrogen‐bonding network, thus endowing aqueous proton batteries with superb rate performance.^[^ ^7^, ^8^ ^]^

Aqueous all‐polymer proton batteries (APPBs) consisting of redox‐active polymer electrodes (RAPs) as the capacity carrying materials are emerging recently.^[^ ^9^ ^]^ By circumventing the crystal lattice collapse problem of inorganic materials and the dissolution issue of organic molecules in electrolytes,^[^ ^10^, ^11^, ^12^, ^13^ ^]^ RAPs offer envisioned benefits such as avoiding expansion of electrode structure and ease of tuning properties via chemical modification, as well as low cost and sustainability. Quinone‐based RAPs have demonstrated suitability for proton cycling and charge storage via the conversion between benzoquinone and hydroquinone (Q/QH_2_).^[^ ^14^, ^15^ ^]^ To tackle challenges of declined capacity stemming from the low electrical conductivity and solubility problems, one promising strategy developed by the Sjödin group is to covalently link redox‐active quinones into conducting polymers (CPs) backbone as pendants forming redox conducting polymers, i.e., Naphthoquinone (NQ) or benzoquinone (BQ) attached to the PEDOT backbone.^[^ ^16^ ^]^ This design facilitates the uptake/removal of protons in addition to the doping/dedoping process of CPs, thus simultaneously harnessing the properties of both the conjugated structure and quinones.

Although quinone‐RAPs have been employed in rocking‐chair proton batteries, APPBs are still in their early stages due to the lack of rational design of organic cathode and anode couples. This is partly caused by the formidable challenges of sustaining a high current rate while maximizing high cell voltage within a narrow electrochemical window in aqueous electrolytes.^[^ ^17^ ^]^ The output voltage for APPBs is determined by the potential difference between the cathode and anode. For anodes, the primary challenge is to develop n‐type quinone‐RAPs that can sustain redox activity as low as the proton reduction potential in aqueous electrolytes. Considering the limited anode potential, organic cathode materials with high potential are essential to enlarge the cell output voltage. Currently developed quinone‐RAPs cathode materials are mainly based on *para*‐quinone (*p*‐Q) building blocks, and their potential need to be promoted.^[^ ^18^ ^]^ Previous attempts have demonstrated that a conducting redox‐active polymer with *p*‐BQ pendants delivers a potential of 0.48 V (vs Ag/AgCl). Couples with NQ‐based anode, the aqueous proton battery could only achieve a voltage of 0.4 V with a capacity of 60 mAh g^−1^.^[^ ^16^ ^]^ In contrast, the bioinspired redox‐active catechol quinones (precursor for *ortho*‐quinone, *o*‐Q) provide strong aromaticity in the reduced state, raising discharged potentials (hence, energy density) and enhancing reaction reversibility.^[^ ^19^ ^]^ The oxidized form is destabilized by the repulsion of two dipoles that direct in similar directions. This will cause significant electron delocalization, resulting in a high potential for the cathode material.^[^ ^21^ ^]^ Motivated by the above‐mentioned merits, Patil et al. have synthesized catechol‐based polyethylene as organic cathodes for Li^+^/H^+^ storage with a high potential of 0.57 V (vs Ag/AgCl).^[^ ^20^ ^]^ To improve the high current rate, pseudocapacitive materials such as conjugated polymer are proposed to store charge through the faraday‐like process, resulting from the fast kinetics and no limitation by semi‐infinite diffusion.^[^ ^22^ ^]^ Therefore, molecular‐level design of pseudocapacitive polymers with low lowest unoccupied molecular orbital (LUMO) energy level and charge separation of frontier molecular orbitals provide another way along the route toward both high voltage (thus, energy/power density) and outstanding rate capability.

Herein, we reported a high‐performance APPBs battery comprising a catechol‐based PEDOT (PTC) cathode and an AQ‐based polyurethane (PUQ) anode. The additive‐free cathode synthesized by grafting PEDOT with caffeic acid pendants showed high redox potential and outstanding rate capability, attributed to the significant electron delocalization and pseudocapacitive‑dominated charge storage. This anode design coupling hard AQ moiety with a soft alkyl chain might alleviate the volume change‐induced electrode rupture, resulting in improved cycling performance. The diffusion‐type PUQ anode shows increased overpotential, making it stable under negative potential. Moreover, the proton storage and anion insertion process in an aqueous electrolyte was validated using DFT calculations and ex situ structural characterizations, providing insights into the molecular‐level design for carbonyl polymer electrodes in rechargeable batteries.

## 2 Results and Discussion

### 2.1 Design Principle of the All‐Polymer Aqueous Proton Battery

**Figure 1:**
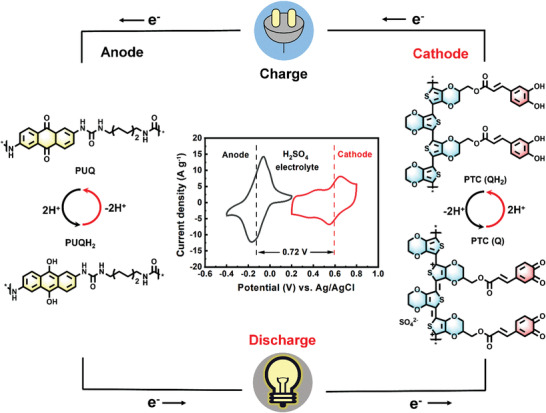
Schematic illustration of polymer electrodes and redox reactions of the all‐polymer proton battery, inset shows the CV curves of polymer electrodes in 0.5 m H_2_SO_4_ electrolyte.

**Figure** **1** shows the working principle and chemical structures of the APPBs composed of quinone‐based polymer electrodes during the charge/discharge process. The proton rocking‐chair cell was based on a two‐electron/proton (2e^−^/2H^+^) process with quinone/hydroquinone (Q/QH_2_) conversion. The cyclic voltammetry (CV) curves showed that the APPBs could deliver a cell voltage of 0.72 V in 0.5 m H_2_SO_4_ electrolytes. Quinone redox moieties (catechol and AQ) were introduced into the side chain of PEDOT and the main chain of PU through electrochemical and chemical polymerization, respectively (Scheme S1, Supporting Information and detailed ^1^H NMR results were shown in Figure S1, Supporting Information). The cathode was prepared via electrochemical copolymerization of EDOT and EDOT‐CQH_2_ monomers on a graphite substrate. During the electropolymerization, the current gradually increased with the growth of polymer segments, yielding a conductive cathode free of any additives (electrical conductivity of 3.6 S cm^−1^, Figure S2a,b, Supporting Information). Apart from the extra capacity afforded by SO_4_^2−^ doping, the PEDOT backbone expedited electron transport and provided pseudocapacitance,^[^ ^23^ ^]^ endowing the cathode with fast charge/discharge behavior. AQ was linked by alkyl chains and anchored in the PU main chain through a chemical polyaddition reaction. This anode design coupled hard AQ moiety with a soft alkyl chain to maintain the crystallinity for proton insertion/extraction and alleviated the rupture of the electrode structure caused by the volume change, thus endowing the anode with stable cycling performance (Figure S2c, Supporting Information).^[^ ^24^ ^]^

### 2.2 Electrochemical Kinetics and Performance of Polymer Electrodes

**Figure 2:**
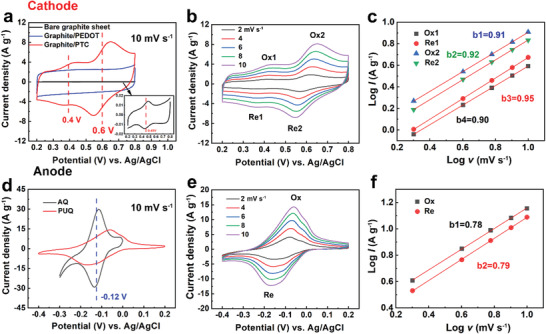
The charge storage kinetics of polymer electrodes in a half‐cell. a) CV comparison of a PTC cathode on graphite, PEDOT on graphite, and bare graphite at 10 mV s^−1^, inset shows the enlarged CV curve of bare graphite. b) CV curves of PTC cathode at different scan rates. c) Fitting curves and *b* values of PTC cathode. d) CV comparison of PUQ and AQ anodes at 10 mV s^−1^. e) CV curves of PUQ anode at different scan rates. f) Fitting curves and *b* values of PUQ anode.

We firstly examined the electrochemical properties of polymer electrodes in 0.5 m H_2_SO_4_ electrolytes. CV curves of PTC cathode on graphite substrates showed two pairs of redox peaks and accompanied with a gradual and slight peak shift. To clarify the attribution of these peaks, CV curves of PEDOT/graphite and bare graphite were also presented as a control in **Figure** **2**a. Peaks at 0.48/0.42 V were observed on bare graphite, attributed to oxygen functional groups on the surface of the graphite sheet.^[^ ^25^ ^]^ Hence, peaks at 0.62/0.57 V versus Ag/AgCl at a scan rate of 2 mV s^−1^ were assigned to the redox reaction of catechol (Figure 2b). It is worth noting that the redox potential of PTC cathode was higher than previously reported CPs grafted by NQ (0.25 V vs Ag/AgCl) and BQ (0.47 V vs Ag/AgCl).^[^ ^16^ ^]^ The charge storage kinetics of PTC cathode was also illustrated at various scan rates in Figure 2b. According to the relationship between peak current densities and scan rates, the *b* values (Figure 2c) were calculated as 0.95/0.90 and 0.91/0.92, respectively, indicating a surface‐controlled dominated charge‐storage process.^[^ ^23^, ^26^ ^]^ The capacitive contribution values (Figure S3a,c, Supporting Information) were determined to be from 82.89% to 91.47% at the scan rates of 2 to 10 mV s^−1^. This high surface‐controlled contribution may be attributed to the collaboration interaction of PEDOT and catechol, reflecting the fast kinetics.

CV curves of PUQ anode in Figure 2d displayed a pair of redox peaks at −0.18/−0.06 V and were stable until −0.40 V. On the contrary, the AQ molecule showed a similar balance potential with higher current density; however, it was unstable beyond ‐0.30 V. Although there was an inevitable capacity loss, an increase in the overpotential introduced by the alky chain slowed down the hydrogen evolution reaction (HER) activity, making the anode more stable under negative potential. Figure S4a in the Supporting Information depicted the HER polarization curves of AQ and PUQ anodes using linear sweep voltammetry (LSV). The PUQ anode showed significantly reduced HER activity, evidenced by the substantially larger onset overpotential of 362 mV than 113 mV for AQ at 10 mA cm^−2^. The reduced HER activity was also demonstrated by the Tafel slope of 197 mV dec^−1^ for PUQ, compared to 77 mV dec^−1^ for AQ (Figure S4b, Supporting Information). Therefore, the PUQ anode displayed similar redox potential to the AQ molecule while also expanding the cell voltage window. The *b* values from the linear fitting curves were 0.78 and 0.79 (Figure 2e,f), indicating a mixed surface and diffusion‐controlled process. Combined with the capacitive contribution results in Figure S3b,d in the Supporting Information (45.96% to 68.53% from 2 to 10 mV s^−1^), PUQ anode was proved to offer both the interaction sites for proton diffusion (at low scan rates) and capacitance (at high scan rates) on the surface. Above all, PTC cathode was a capacitive‐type electrode while PUQ anode was more like a hybrid electrode so that the improvement can be realized both in energy density and power density.

**Figure 3:**
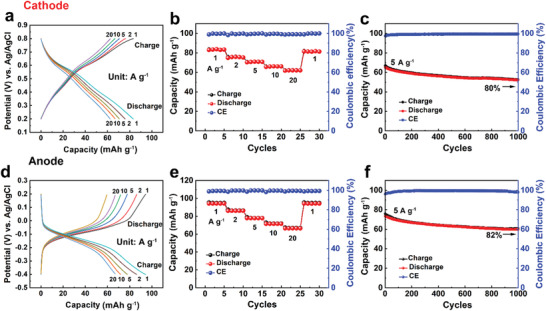
Electrochemical performance of polymer electrodes in 0.5 m H_2_SO_4_ electrolyte. GCD curves of the cathode a) and anode d) at different current densities; C‐rate and coulombic efficiency of the cathode b) and anode e). The capacity retention of the cathode c) and anode f) during 1000 cycles at 5 A g^−1^.

The specific capacity and stability of polymer electrodes were evaluated by galvanostatic charge–discharge (GCD) tests at various current densities. Owing to the stable conjugated backbone, PTC cathode underwent a reversible redox process and gave a notably discharged capacity of 83.2 mAh g^−1^ at 1 A g^−1^ and 62.0 mAh g^−1^ was retained at 20 A g^−1^ (**Figure** **3**a,b), with a nearly 100% coulombic efficiency (CE) at varied current rates. PTC cathode exhibited excellent capacity retention (74.5%) at a high current rate of 20 A g^−1^, attributed to the pseudocapacitance provided by the PEDOT backbone. When the current density was reverted to 1 A g^−1^, the cathode still possessed nearly the same capacity as the pristine state, demonstrating excellent reversibility. Besides, the differential capacity versus voltage plot (d*Q* d*V* ^−1^) was carried out to explore the source of capacity (Figure S5a, Supporting Information).^[^ ^27^, ^28^ ^]^ An evident quick rise appeared at around 0.60 V, corresponding to the redox reaction of catechol, which was consistent with the CV results.

PUQ anode delivered a discharged capacity of 94.2 to 66.5 mAh g^−1^ from 1 to 20 A g^−1^ and recovered to 94 mAh g^−1^ when the current density returned to 1 A g^−1^, demonstrating good reversibility and retention to a high current rate (Figure 3d,e). Unlike the pseudocapacitance from PTC cathode, the capacity source of PUQ anode mainly came from the redox process of AQ sites, as evidenced by the redox peaks from the d*Q* d*V* ^−1^ curve shown in Figure S5b in the Supporting Information. The peak was so sharp that an evident potential plateau was observed in the GCD curves, and the platform became steeper as the current density increased (Figure 3d). Moreover, both the anode and cathode gave a stable cycling performance (≈80% capacity retention) for at least 1000 cycles at 5 A g^−1^ (Figure 3c,f), indicating that the molecular anchoring strategy effectively suppressed the dissolution of quinone redox‐active materials. Besides, the introduction of an alkyl chain increased the flexibility of PUQ to accommodate the volume expansion during the charge/discharge processes, enabling the anode with excellent stability.^[^ ^29^ ^]^

### 2.3 Ex Situ Characterizations and DFT Calculations of Polymer Electrodes

**Figure 4:**
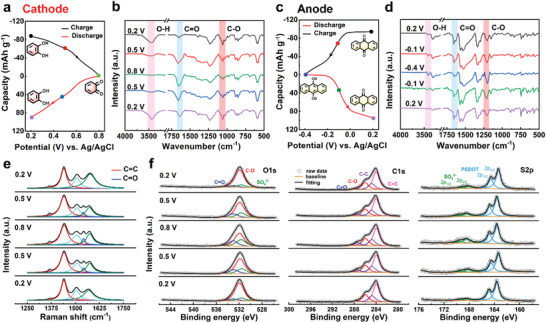
Structural characterizations of polymer electrodes during different charge/discharge states. The ex situ FT‐IR change at different potentials of the cathode a,b) and anode c,d); the Raman e) and XPS spectra of the O 1s, C 1s, and S 2p region f) of the cathode.

To reveal the electrochemical reaction mechanism of polymer electrodes, various ex situ characterizations were carried out to identify the role of quinone redox moieties. **Figure** **4**a,b showed the FT‐IR and corresponding structure changes of PTC cathode under different charge/discharge states. When the cathode was charged from 0.2 to 0.8 V, the band around 1680–1730 cm^−1^ (assigned to stretching vibrations of C═O bonds) gradually increased, while the band located at 3350–3500 and 1050–1100 cm^−1^ (assigned to stretching vibration of —OH and C—O) became weaker, indicating the transition from QH_2_ to Q.^[^ ^24^, ^30^ ^]^ In the reversed discharge process, the —OH and C—O absorption bands recovered to their original intensity, implying a reversible arrangement of catechol from Q to QH_2_. The Raman spectrum in Figure 4e also proved the structural change of PTC cathode. Raman shift at 1560 cm^−1^ of C═O increased sharply compared to the shift at 1520 cm^−1^ of C═C during the charging process.^[^ ^30^ ^]^ The absorption intensity of C═O bonds decreased when PTC was discharged to 0.2 V, consistent with the FT‐IR results. The structure changes of PUQ anode were also characterized by the FT‐IR spectra in Figure 4c,d. The results showed a comparable changing trend, indicating a similar redox mechanism of *p*‐Q and *o*‐Q structures. During the charging process, the absorption intensity of C═O decreased while the absorption intensity of C—O and O—H groups rose from 0.2 V to —0.4 V. In the reversed discharge process, peak intensities showed the opposite change trend.

To further explore the charge/discharge mechanism of PTC cathode, the binding energy of O 1s, C 1s, S 2p, and their oxidation states was analyzed by ex situ X‐ray photoelectron spectroscopy (XPS, Figure 4f). In the O 1s region, the ratio of peaks at 533.1 to 532 eV (corresponding to C═O and C—O) became stronger during the charging process and reached the maximum at 0.8 V. It declined in the discharged state, proving a reversible redox reaction. The relative intensity of the C═O peak (287.5 eV) in the C 1s region increased in the charged state and decreased in the discharged state. These findings, along with the FTIR and Raman peak alterations, showed the proton storage mechanism of catechol. Beyond that, the immobilization of SO_4_^2−^ anion also contributed to the capacity related to the doping/dedoping of the PEDOT backbone. In the S 2p region, the strong peaks (2p_1/2_, 169.5 eV; 2p_3/2_, 168.5 eV) in the charging process proved the insertion of SO_4_^2−^ anion, which was consistent with SO_4_^2−^ (531.6 eV) alterations in O 1s region. Meantime, there was offset toward high binding energy in PEDOT (2p_1/2_, 2p_3/2_) during the charge process owing to the increase in positive charge. Taken together, the electrochemical reaction mechanism of PTC cathode was dominated by the dual ions (H^+^ and SO_4_^2−^) insertion in the aqueous electrolyte.

**Figure 5:**
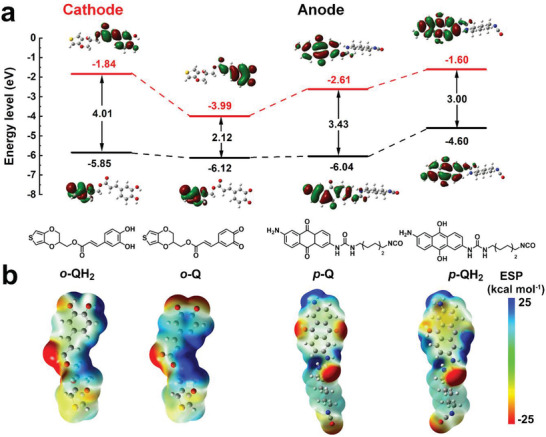
Theoretical modeling of monomers with DFT calculation. a) Chemical structures and calculated energy levels of *o*‐Q/*o*‐QH_2_ (PTC cathode), *p*‐Q/*p*‐QH_2_ (PUQ anode), respectively. b) Electrostatic potential (ESP) and charge distribution of the monomers. The blue and red surface represents the highest (fewer electrons) and lowest (more electrons) electrostatic potential.

The impact of quinone redox moieties (catechol and AQ) on the electronic structure of two monomers was investigated with DFT calculation. The theoretical calculation of the molecular orbitals is shown in **Figure** **5**a. Complete separation of the highest occupied molecular orbital (HOMO) and the LUMO was observed on the EDOT‐CQ monomers. Whether it was in the oxidized (EDOT‐CQ) or reduced state (EDOT‐CQH_2_), the electron cloud of the HOMO orbital was distributed on the EDOT moiety while that of the LUMO orbital was on the pendant catechol moiety, indicating the potential of the cathode was related to the catechol pendants. This structure was also beneficial to the charge transport since there was an intramolecular charge transfer effect between catechol and EDOT backbone, where electrons flowed rapidly among the polymer.^[^ ^31^ ^]^ Unlike the EDOT‐CQ monomers, the HOMO/LUMO orbitals of PUQ segments concentrated on the AQ moiety, and there was no electron cloud distributed on the alkyl chain. Therefore, only AQ moiety was responsible for the redox reactions, making PUQ anode closer to intercalation behavior due to its crystallization.

Frontier orbital energy levels were also calculated using DFT calculation, in which the discharging potential was associated with the LUMO levels of electrode materials.^[^ ^32^, ^33^ ^]^ The LUMO level of the oxidized PTC cathode bearing *o*‐Q moiety (−3.99 eV) was deeper than that of the oxidized PUQ anode bearing *p*‐Q moiety (−2.61 eV), indicating a higher reduction potential. In contrast, the reduced *o*‐QH_2_ moiety showed a deeper‐lying HOMO level (−1.84 eV) than the *p*‐QH_2_ moiety (−1.60 eV), implying a higher oxidation potential. Additionally, PTC cathode showed a narrower bandgap (2.12 eV) than PUQ anode (3.43 eV), attributed to the stronger electron‐withdrawing ability of catechol pendants. Subsequently, the reaction site of electrodes was imitated using molecular electrostatic potential from DFT calculations. Both the anode and cathode material showed similar results in Figure 5b. The oxygen atoms in the oxidized Q state showed a negative electrostatic potential, indicating strong electron‐donating capacity and readiness to absorb protons, corresponding to the conversion from C═O to C—OH. On the contrary, the oxygen atoms in the reduced QH_2_ state showed positive electrostatic potential, corresponding to the conversion from C—OH to C═O.^[^ ^4^, ^24^ ^]^

In order to reveal the active sites more intuitively, we presented the charge distribution of the two monomers under oxidation states (Figure S6, Supporting Information). The negative charge was concentrated mostly on the oxygen and nitrogen atoms, as predicted. For *o*‐Q, the oxygen atoms on the EDOT ring and the ester group showed no redox activity. For *p*‐Q, the amino group reacted with the isocyanate to generate an inactive urea bond after polymerization. Therefore, the proton‐coupled redox reactions were more likely to occur on oxygen atoms with high electronegativity in the benzene and anthracene rings. Overall, the charge storage mechanism of quinone‐based polymer electrodes containing *o*‐Q and *p*‐Q structures were validated by DFT calculations together with various ex situ structural characterizations.

### 2.4 Electrochemical Performance of the PTC‐PUQ Aqueous Proton Battery

**Figure 6:**
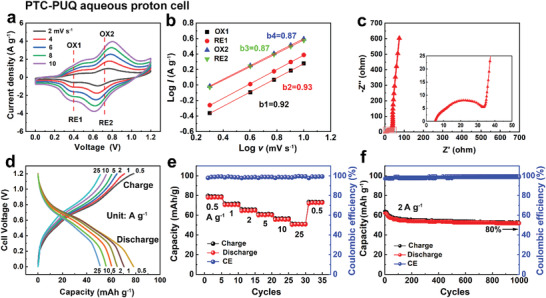
Electrochemical behavior of the PTC‐PUQ aqueous proton battery in 0.5 m H_2_SO_4_ electrolyte. a) CV curves at various scan rates; b) *b* values fitting from the peak currents; c) EIS of the aqueous proton cell at the open‐circle potential; d) GCD curves at different current densities; e) C‐rate performance and coulombic efficiency; f) capacity retention after 1000 cycles at 2 A g^−1^.

Based on the potential difference between PTC cathode and PUQ anode (Figure 1; Figure S7, Supporting Information), a Swagelok cell was assembled using 0.5 m H_2_SO_4_ electrolyte. The all‐polymer proton battery exhibited a cell voltage of 0.72 V as shown in CV curves. It is evident that two redox peaks appeared at 0.40 and 0.72 V (**Figure** **6**a). During the discharged process, the C—OH groups of PUQ were oxidized to C═O, while the C═O bonds of PTC were simultaneously reduced to C—OH groups accompanied by the absorption of protons. The reverse redox reactions occurred in the charged process with the opposite structure changes. We fitted the linear relationship between the scan rates and peak current densities and calculated *b* values to be 0.92/0.93 (Ox_1_/Re_1_) and 0.87/0.87 (Ox_2_/Re_2_) (Figure 6b), indicating a capacitive‐controlled process. The faradaic charge storage process without diffusion limitation was beneficial to the high capacity retention at a high rate.^[^ ^22^ ^]^

The electrochemical performance was further revealed by the electrochemical impedance spectroscopy (EIS) using Randle's type circuit model shown in Figure 6c.^[^ ^34^, ^35^ ^]^ In the low‐frequency regions, the line of PTC cathode (80°) was more vertical than that of PUQ anode (50°), evidencing more capacitive behaviors (Figure S8, Supporting Information). The charge transport resistance (*R*_ct_) can be evaluated by fitting the semicircle curve in the high‐frequency regions. The *R*_ct_ of the full proton cell was 30 Ω, and the line in the low‐frequency regions was kept vertical (75°). The proton cell delivered a high specific capacity of 78.1 to 50.8 mAh g^−1^ at discharge densities from 0.5 to 25 A g^−1^ and was accompanied by a voltage plateau between 0.3 and 0.9 V (Figure 6d). Attributed to the pseudocapacitive‐controlled process of PTC cathode, the proton cell exhibited excellent rate capability and CE (Figure 6e).^[^ ^36^ ^]^ The proton cell retained 50.8 mAh g^−1^ (65.0% of pristine capacity retention) at the high current rate of 25 A g^−1^. Moreover, Figure 6f showed the capacity retention was 80% after 1000 cycles at 2 A g^−1^, with a high CE (≈98%).

**Figure 7:**
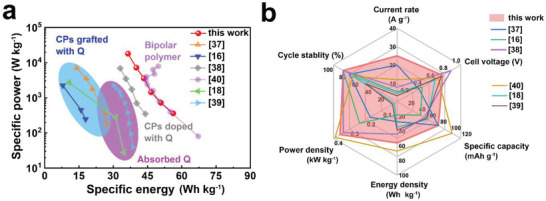
The electrochemical performance comparison. a) Ragone plot and b) Radar chart of the electrochemical performance among reported all‐organic aqueous proton batteries. Cycles stability was evaluated by the capacity retention after 500 GCD cycles; the current rate was the highest current density that the proton cell could bear.

As shown in the Ragone curves (**Figure** **7**a, detailed results were listed in Table S1 in the Supporting Information), the full‐organic proton cell offered a high cell voltage (0.72 V), high specific capacity (78.1 mAh g^−1^), energy density (56.2 Wh kg^−1^), power density (360 W kg^−1^) and superior rate capability (51.2 mAh g^−1^ at 25 A g^−1^). This performance was superior to the most reported aqueous organic proton cells, including CPs grafted by quinones (pDTP‐AQ/pDTP‐NQ couple,^[^ ^37^ ^]^ pEP(NQ)E/pEP(QH_2_)E couple^[^ ^16^ ^]^), CPs doped with quinones (Perylene‐PI/PEDOT‐lignin couple,^[^ ^38^ ^]^ and quinones molecule absorbed electrodes (AQ/TCHQ couple,^[^ ^18^ ^]^ AQDS‐CC/Tiron‐CC couple^[^ ^39^ ^]^). High cell voltage and rate capacity were achieved by the charge separation of PEDOT and catechol pendants. Although the specific capacity and cell voltage were slightly inferior to those of the bipolar molecules such as PNAQ, it should be noted that the proton cell held better cyclic stability (85% capacity retention over 500 cycles) than that of PANQ^[^ ^40^ ^]^ (70% capacity retention over 500 cycles) in the Radar chart (Figure 7b). SEM images were also performed to study the morphology stability of polymer electrodes before and after 1000 cycles. Both the cathode and anode surfaces remained pristine smooth, and uniform morphology after long‐term cycles (Figure S9, Supporting Information). PTC cathode was composed of particles/aggregates, while PUQ anode showed nanosphere morphology with 30–60 nm diameter. The highly stable pseudocapacitive PTC cathode and flexible PUQ anode with robust morphology contributed to the superior cycling stability.

## 3 Conclusion

In this work, we have developed an all‐polymer aqueous proton battery with superior electrochemical performance using the catechol‐based cathode and AQ‐based anode. A high potential cathode has been realized by introducing catechol pendants to the PEDOT backbone, which provides significant electron delocalization. The electron cloud separation of redox pendants and conjugated backbone benefited the pseudocapacitive charge storage, enabling excellent rate performance. The diffusion‐type anode with rigid and soft segments could increase the overpotential and reduce the volume change‐induced electrode rupture, thereby promoting cycling stability. The DFT calculation and the ex situ structural changes might deepen the understanding of the proton storage mechanism in quinone‐based polymer electrodes. Combining the capacitive‐type cathode and diffusion‐type anode, the aqueous proton cell possessed high voltage, excellent energy/power densities, and superior rate capacity. These results highlight the feasibility of rational design of high‐performance polymer electrodes for sustainable energy storage systems.

## 4 Experimental Section

4.1

#### Materials Synthesis; Synthesis of EDOT‐MeCl

Firstly, 3,4‐dimethoxythiophene (1.9 mL, 15.8 mmol), 3‐chloro‐1,2‐propanediol (3.72 mL, 44.4 mmol), and *p*‐toluenesulfonic acid monohydrate (0.31 g, 1.6 mmol) were added into 50 mL of dry toluene solvent. The mixed solution was continuously stirred at 100 °C for one day. Then, another 3.72 mL (22.2 mmol) of diol were added, and the mixture was heated at 100 °C for another 3 h. After removing toluene, the remaining crude product was purified by flash chromatography (silica gel, hexane:dichloromethane = 4:2) to obtain a white solid substance, EDOT‐MeCl. ^1^H NMR (400 MHz, CDCl_3_, *δ*): 6.39‐6.33 (m, 2H), 4.40‐4.33 (m, 1H), 4.27 (d, J = 11.7 Hz, 1H), 4.19‐4.11 (m, 1H), 3.76‐3.62 (m, 2H) (Figure S1a, Supporting Information).

#### Synthesis of EDOT‐CQH_2_

NaHCO_3_ (1.80 g, 21.04 mmol) and caffeic acid (1.89 g, 10.52 mmol) was firstly added into the flask, and DMF (50 mL) was then added. Subsequently, EDOT‐MeCl (1.00 g, 5.26 mmol) was added to the above solution. The solution was stirred at 100 °C for three days under the protection of nitrogen. After that, the solution was cooled down to room temperature; the mixture was poured into water and extracted with EtOAc several times. The combined organic phase was sequentially washed with saturated NaHCO_3_, NaCl solutions and then dried with anhydrous MgSO_4_. After the organic solvent was removed under vacuum, the crude product was further purified by flash column chromatography on silica (hexane: EtOAc = 8:1), giving the EDOT‐CQH_2_ (1.2 mmol, 0.36 g, 24%) as a colorless viscous liquid. ^1^H NMR (400 MHz, DMSO‐*d*_6_, *δ*): 9.11 (s, 1H), 9.00 (s, 1H), 6.96‐6.88 (m, 2H), 6.83 (dd, J = 8.3, 2.0 Hz, 1H), 6.63‐6.61 (m, 2H), 5.64‐5.55 (m, 1H), 5.13‐5.06 (m, 1H), 4.58‐4.51 (m, 1H), 4.41 (dd, J = 11.7, 2.2 Hz, 1H), 4.28‐4.17 (m, 3H) (Figure S1b, Supporting Information).

#### Synthesis of PTC

3,4‐Ethylenedioxythiophene (EDOT) monomer was used to copolymerize with EDOT‐CQH_2_ at the molar ratio of 1:1. The PTC polymer was electropolymerized using the CV method (20 cycles, 100 mV s^−1^, −0.6 to +1.8 V vs Ag/Ag^+^) from a solution of 10 × 10^−3^ m EDOT and EDOT‐CQH_2_ in 0.1 m TBAPF_6_ in MeCN.

#### Synthesis of PUQ Powder

It was synthesized through the chemical polyaddition reaction. Firstly, 2,6‐diaminoanthraquinone (5 mmol, 1.19 g) and 1,6‐hexanediisocyanate (0.84 g, 5 mmol) were dissolved in 10 mL NMP and reacted at 80 °C for two days. After cooling, the resulting polyurethane was precipitated into diethyl ether and washed with methanol, finally dried under vacuum at 80 °C overnight obtaining an orange solid.

#### Materials Characterizations

The ^1^H nuclear magnetic resonance (NMR) was performed on a 400 MHz NMR spectrometer (Bruker AMX 400 system) to confirm the chemical structures of EDOT‐MeCl and EDOT‐CQH_2_ monomer. Ex situ FT‐IR spectra (BRUKER VECTOR 22 Spectrometer) were used to characterize the structure changes of anodes and cathodes during different charged/discharged stages. Raman spectra (Renishaw's InVia Raman Microscopes) and XPS (Thermo ECSALAB 250) were also used to clarify the reaction mechanism of the cathode, accompanied by C1s peak calibration (284.8 eV). Crystal structures of PUQ powder and anode were characterized using Rigaku wide‐angle X‐ray diffractometer (XRD, D/max rA, using Cu K*α* radiation, *λ* = 1.5406 Å). Morphology was observed using field‐emission SEM (FEI Nova Nano SEM 450) before and after long‐term cycles.

#### Preparation of the Cathode and Anode

PTC cathode was electropolymerized onto the graphite sheet with the mass loading of 1 mg cm^−2^. The as‐prepared polymer was directly used as the cathode without further processing. As the control experiments, PEDOT/graphite sheet and bare graphite were tested with the CV method. The anode was prepared by mixing PUQ powders, acetylene carbon, and PTFE into a homogeneous slurry at a mass ratio of 7:2:1, coated onto a carbon cloth. After drying in a vacuum oven overnight at 60 °C, the anode was loaded with an average mass of 1.5 mg cm^−2^. The AQ electrode was prepared as the control experiment by mixing the AQ power with acetylene carbon and PTFE and coated onto a carbon cloth.

#### Fabrication of the Aqueous All‐Organic Proton Cell

According to the formula, *C*_+_ × *m*_+_ = *C*_−_ × *m*_−_ (*C* and *m* were the actual capacity mass of active material), the mass ratio of anode/cathode materials was 1.05:1 to compensate charge. Before assembling the battery, two electrodes were activated by applying the constant potential, where the anode was reduced to PUQH_2,_ and the cathode was oxidized to PTC(Q). After that, two electrodes were assembled into a Swagelok battery, containing Celgard2400 as the separator and 0.5 m H_2_SO_4_ as the electrolyte.

#### Electrochemical Measurements

The conductivity of cathode, anode, AQ, and PUQ powder was measured by RTS8 four‐probe resistivity tester. Three electrode tests were performed on a CHI660e electrochemical workstation, including CV, GCD, and EIS, with a Pt wire as the counter electrode and an Ag/AgCl electrode as the reference electrode. The protonic electrolyte was bubbled with nitrogen to remove oxygen before the tests. To assess the HER activity of AQ and PUQ electrodes, LSV tests were carried out in N_2_‐bubbled 0.5 m H_2_SO_4_ aqueous electrolyte based on the above three‐electrode configuration. All potentials are calibrated relative to the reversible hydrogen electrode (RHE, 0 V). To eliminate reduction current from anodes, electrodes were exposed to −0.2 V versus Ag/AgCl for 2 h to ensure complete reduction. Galvanostatic specific capacity and capacity retention of the full cell was carried on the Neware battery tester.

The theoretical capacity of the anode and cathode was calculated using the following Equation (1)

(1) $$Qs=\frac{n\times F}{3.6\times M}$$

where *Q*s is the theoretical specific capacity (mAh g^−1^), *n* is the theoretical electron‐transfer numbers of active materials, *F* is the Faraday constant (96 485 C mol^−1^) and *M* is the corresponding molecular mass of polymer fragments. The theoretical capacity of the anode was 135 mAh g^−1^. The cathode capacity was estimated based on the average molecular mass of EDOT and EDOT‐CQH_2_. Thus, the theoretical capacity of the cathode is 225 mAh g^−1^.

To confirm the charge storage kinetics, the energy storage behavior can be revealed by Equation (2)

(2) $$i=a\times v^{b}$$

where *i* was the peak current (A g^−1^) from the CV curve and *v* was the scan rate (mV s^−1^). To further fit the curve, the equation can be written as Equation (3)

(3) $$Log\left(i\right)=b\times Log\left(v\right)+Log\left(a\right)$$

The *b* value was usually in the range of 0.5–1. When *b* approaches 0.5, it is dominated by the diffusion‐control process, which is close to a battery. When *b* approaches 1, it is dominated by the surface‐control process, which is close to a capacitor.

The capacitive contribution (CC) can be calculated by the following Equations (4) and (5)

(4) $$i=k_{1}\times v+k_{2}\;\times v^{\left(1/2\right)}$$

(5) $$\mathrm{CC}=\frac{k_{1}\;\times v}{i}\times 100\%$$

where *i* is current (A g^−1^) at different potentials, *k*_1_ *v* is the capacitive contribution and *k*_2_ *v* ^1/2^ is the ionic diffusion contribution.

The potential of the electrode (*E*_1/2_, *V*) and *V*_cell_ (*V*) were calculated according to Equations (6) and (7)

(6) $$E_{1/2}=\;\left(E_{\mathrm{ox}}+E_{\mathrm{re}}\right)/2$$

(7) $$V_{\mathrm{cell}}=E_{1/2},\;\mathrm{cathode}-E_{1/2},\;\mathrm{anode}$$

where *E*_ox_ and *E*_re_ were the potentials of the oxidation peak and reduction peak, respectively.

The energy density (Wh kg^−1^) and power density (W kg^−1^) were calculated by the following Equations (8) and (9)

(8) $$W=C\times V_{\mathrm{cell}}$$

(9) $$P=I\times V_{\mathrm{cell}}$$

where *C* represents the specific capacity of the full cell, *V*_cell_ represents the cell output voltage, *I* represents the current density. It should be noted that the gravimetric energy/power density was evaluated based on the total mass of the anode and cathode.

#### Theoretical Calculation

DFT calculations were performed in the Gaussian 09 software package to gain structural information of the oxidized/reduced molecules. Geometrical optimization adopted the B3LYP/6‐31G(d) basis sets. Based on the optimized molecular structures, the HOMO/LUMO and electrostatic potential (ESP) analysis were visualized with Gaussview 5.0. All basis sets are obtained from the Basis Set Exchange library. The PTC cathode was truncated to EDOT monomer grafted by catechol (*o*‐QH_2_). For PUQ anode, segments containing AQ and an alkyl chain were employed to represent the *p*‐Q and reduced *p*‐QH_2_ in the same methods. The detailed calculation results can be found in the appendix.

## Conflict of Interest

The authors declare no conflict of interest.

## Supporting information

Supporting Information

## Data Availability

The data that support the findings of this study are available from the corresponding author upon reasonable request.
