# Supplementary material for: Bioinspired Catechol‐Grafting PEDOT Cathode for an All‐Polymer Aqueous Proton Battery with High Voltage and Outstanding Rate Capacity
Source: Adv Sci (Weinh). 2021 Dec 16;9(4):2103896. doi: 10.1002/advs.202103896 (PMC8811804; doi:10.1002/advs.202103896)
Supplement: Supplementary file 1 — Supporting Information [file ADVS-9-2103896-s001.pdf]

ADVANCED SCIENCE
Open Access

Supporting Information

for *Adv. Sci.*, DOI: 10.1002/advs.202103896

Bioinspired catechol-grafting PEDOT cathode for an all-polymer aqueous proton battery with high voltage and outstanding rate capacity

Meihua Zhu, Li Zhao, Qing Ran, Yingchao Zhang, Runchang Peng, Geyu Lu, Xiaoteng Jia, Danming Chao*, Caiyun Wang*

Supporting Information

Bioinspired catechol-grafting PEDOT cathode for an all-polymer aqueous proton battery with high voltage and outstanding rate capacity

Meihua Zhu, Li Zhao, Qing Ran, Yingchao Zhang, Runchang Peng, Geyu Lu, Xiaoteng Jia, Danming Chao*, Caiyun Wang*

M. Zhu, Y. Zhang, Prof. D. Chao

College of Chemistry, Jilin University, Changchun 130012, China

L. Zhao, R. Peng, Prof. G. Lu, Prof. X. Jia

State Key Laboratory of Integrated Optoelectronics, College of Electronic Science and Engineering, Jilin University, Changchun 130012, China

Q. Ran

Key Laboratory of Automobile Materials, Ministry of Education, School of Materials Science and Engineering, Jilin University, Changchun 130022, China

Prof. C. Wang

ARC Centre of Excellence for Electromaterials Science, Intelligent Polymer Research Institute, AIIM Facility, University of Wollongong, Wollongong, NSW 2522, Australia

Email: xtjia@jlu.edu.cn (X.J.), chaodanming@jlu.edu.cn (D.C.)

Keywords: Polymer electrode, All-organic battery, Catechol, PEDOT, Aqueous proton battery

Table S1. Electrochemical performance comparison of PTC-PUQ cell with advanced all-organic aqueous proton batteries.

Anode/Cathode	Voltage[1] (V)	Specific capacity (mAh/g @ A/g)	Energy density[2] (Wh/kg)	Power density[2] (kW/kg)	Cycle stability[3] (retention after 500 cycles)	Current rate[4] (A/g)	Ref.
PUQ/PTC	0.72	78.1, @0.5	56.2	360	85%	25	This work
pDTP-AQ/ pDTP-NQ	0.35	78, @0.5	27.3	175	75%	20	[18]
pEP(NQ)E/ pEP(QH_2)E	0.4	45, @0.6	18	240	85%	5.5	[17]
Perylene-PI/ PEDOT-lignin	0.85	52, @0.4	44.2	340	85%	8	[38]
PNAQ/PNAQ	0.65	103.7, @0.12	67.4	78	70%	12	[40]
AQ/TCHQ	0.65	58.1, @0.045	37.75	29.25	70%	4.5	[20]
AQDS-CC/ Tiron-CC	0.7	55.4, @0.055	38.8	38.5	50%	3.3	[39]

Notes:

[1] Voltage was the potential difference between the cathode and anode;

[2] Energy density and power density were calculated from the maximum specific capacity;

[3] Cycles stability was evaluated by the capacity retention after 500 GCD cycles;

[4] Current rate was the highest current density the proton cells bear.

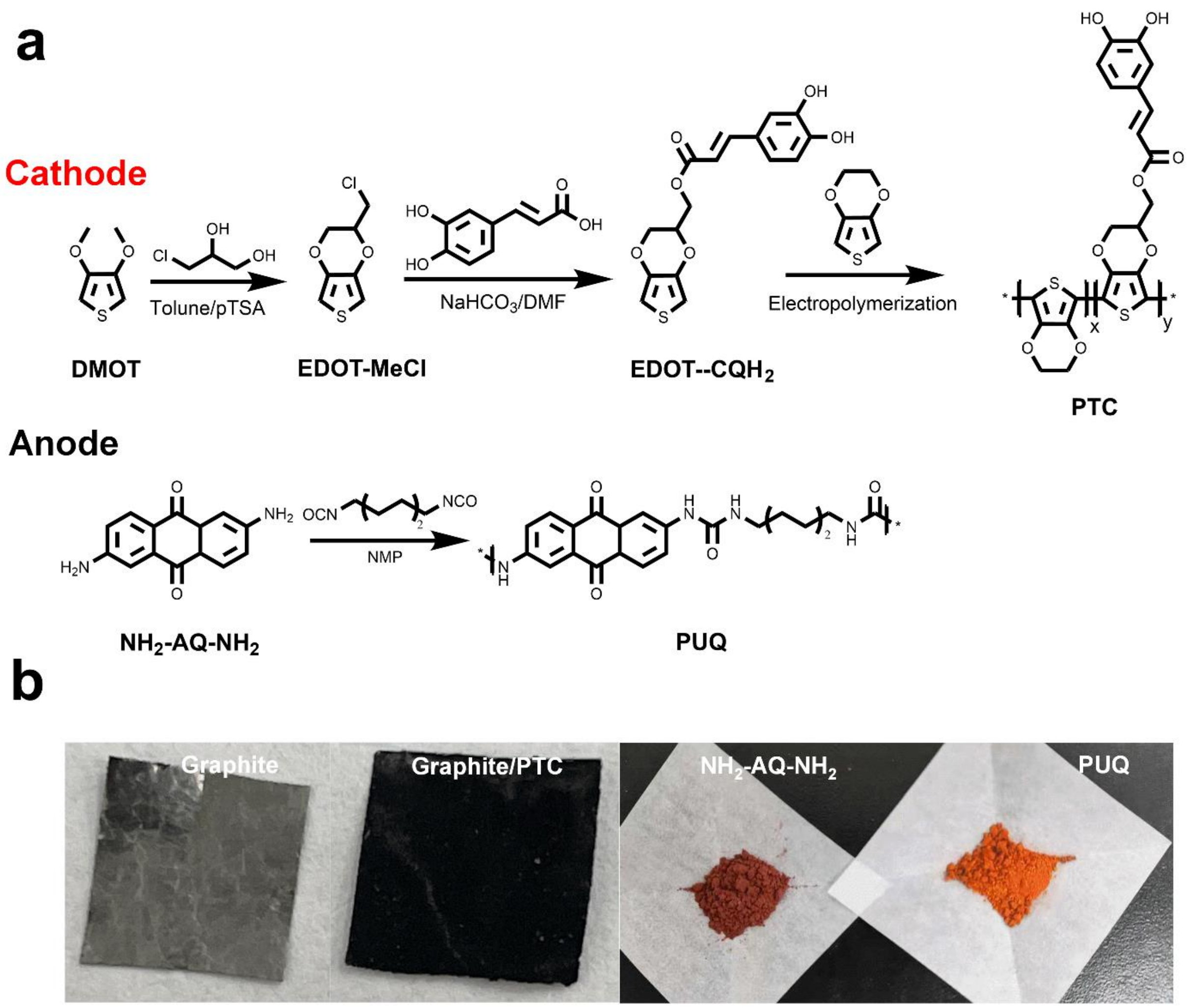

Scheme S1. (a) Synthetic process and (b) photographs of PTC on graphite and PUQ powder.

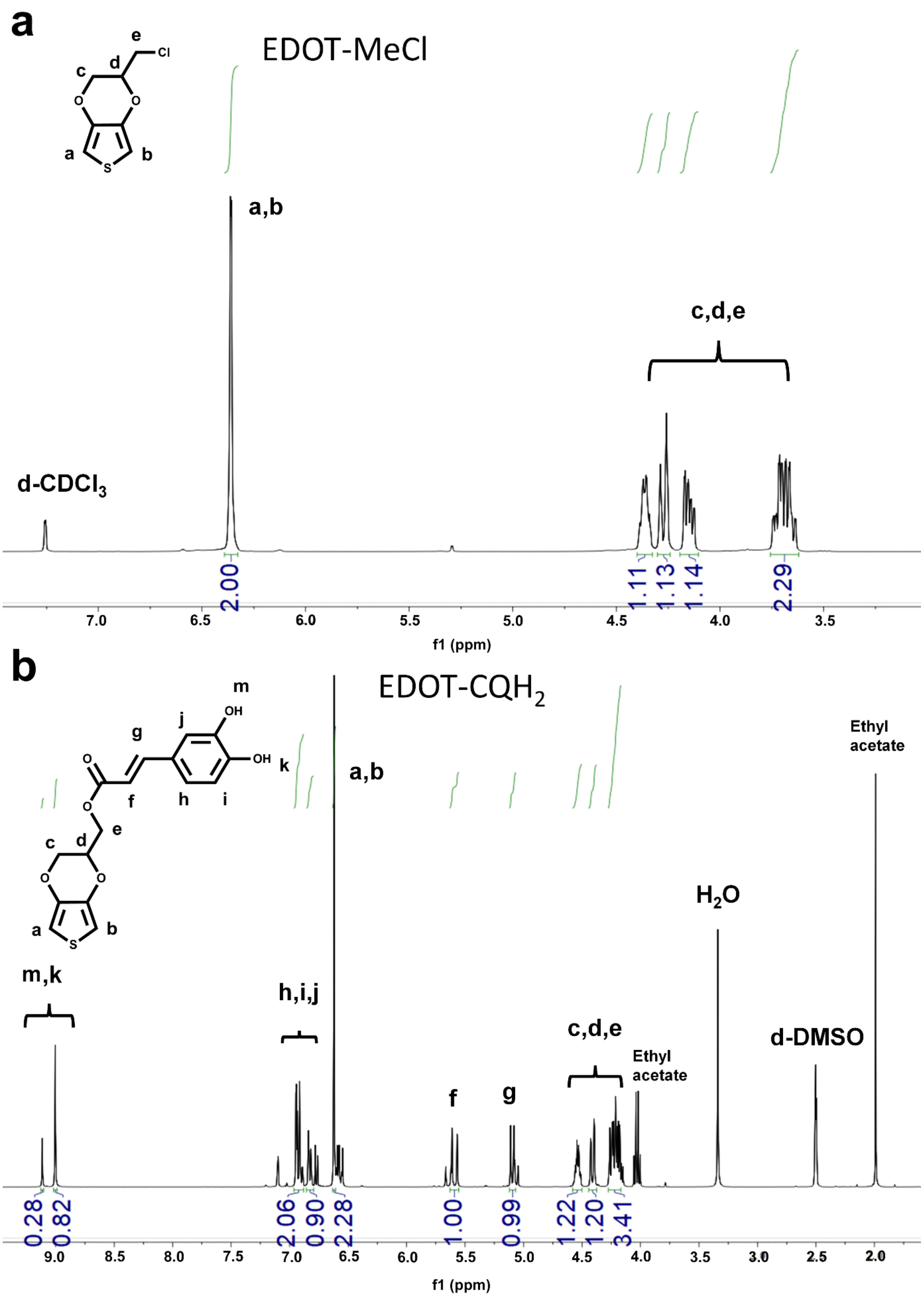

Figure S1. ^{1}H NMR of (a) EDOT-MeCl in d-$CDCl_3$ and (b) EDOT-CQH_2 in DMSO-d_6.

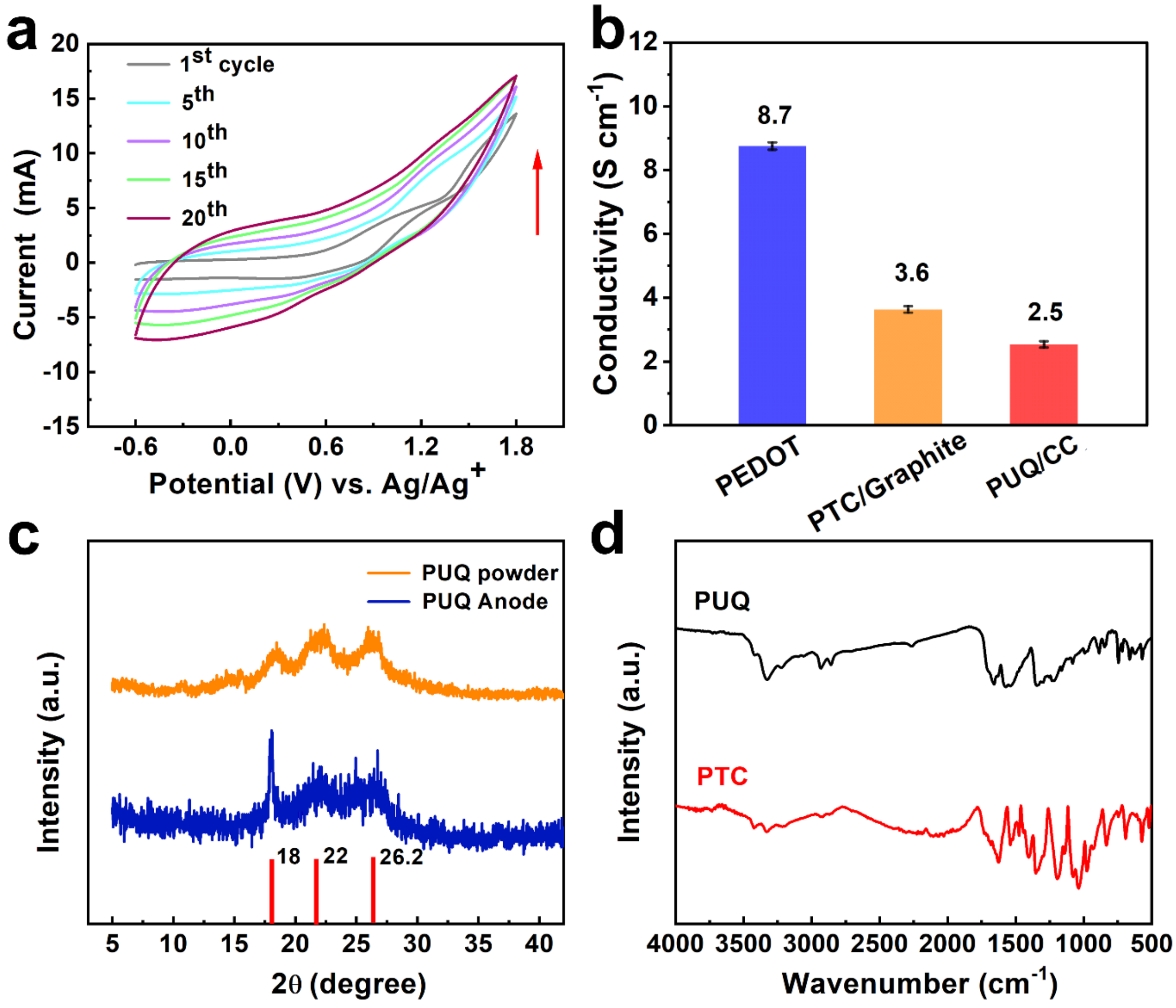

Figure S2. (a) CV of PTC on graphite during the electrochemical polymerization; (b) Electrical conductivity of the PEDOT/graphite, PTC/graphite cathode, PUQ/carbon cloth anode; (c) XRD curves of the PUQ powder and PUQ/carbon cloth anode; (d) FT-IR spectra of the PUQ and PTC polymers.

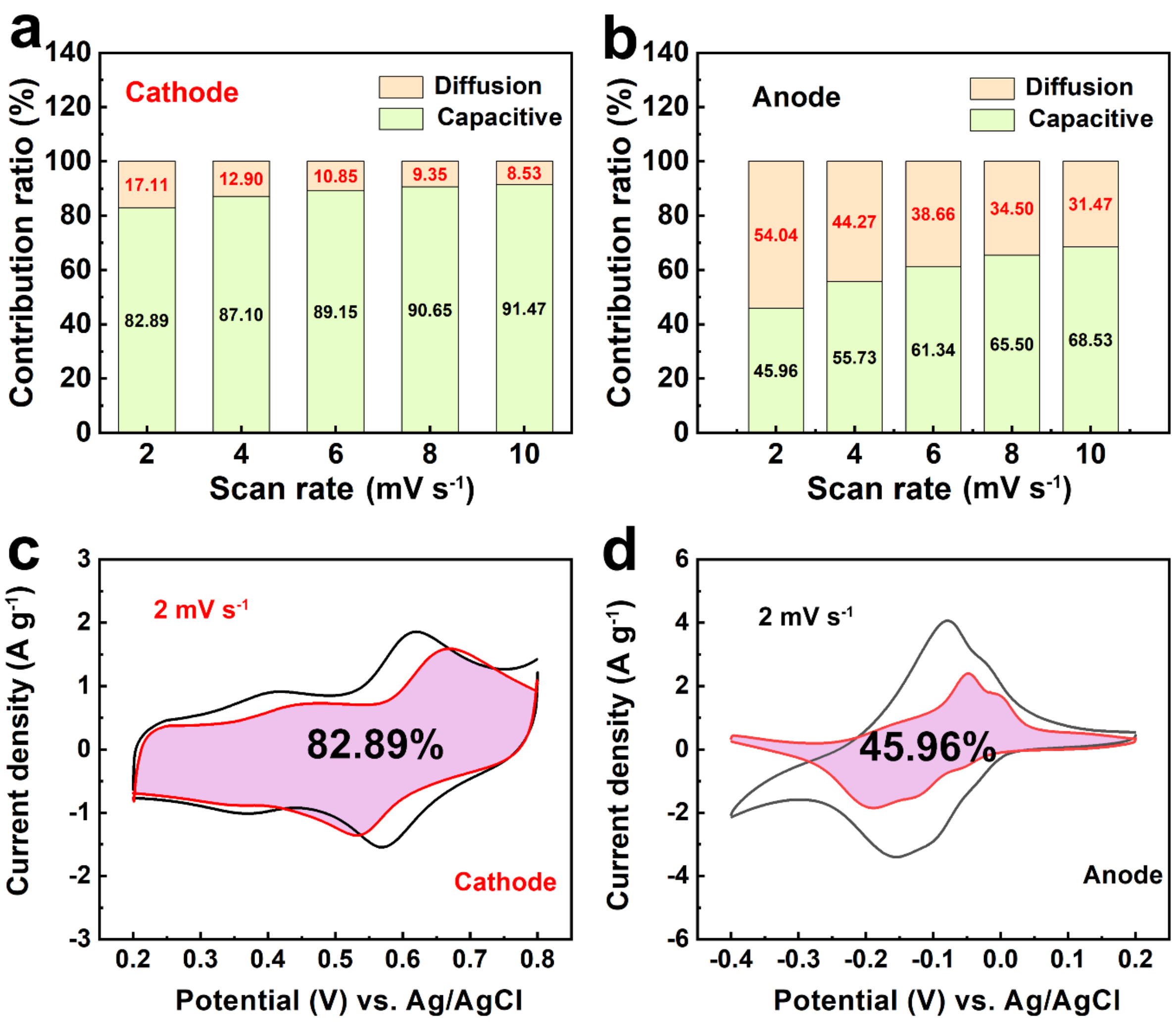

Figure S3. Capacitive contributions of the cathode (a) and anode (b) at various scan rates; Fitting capacitive curve and pristine CV curves of the cathode (c) and anode (d) at 2 mV/s.

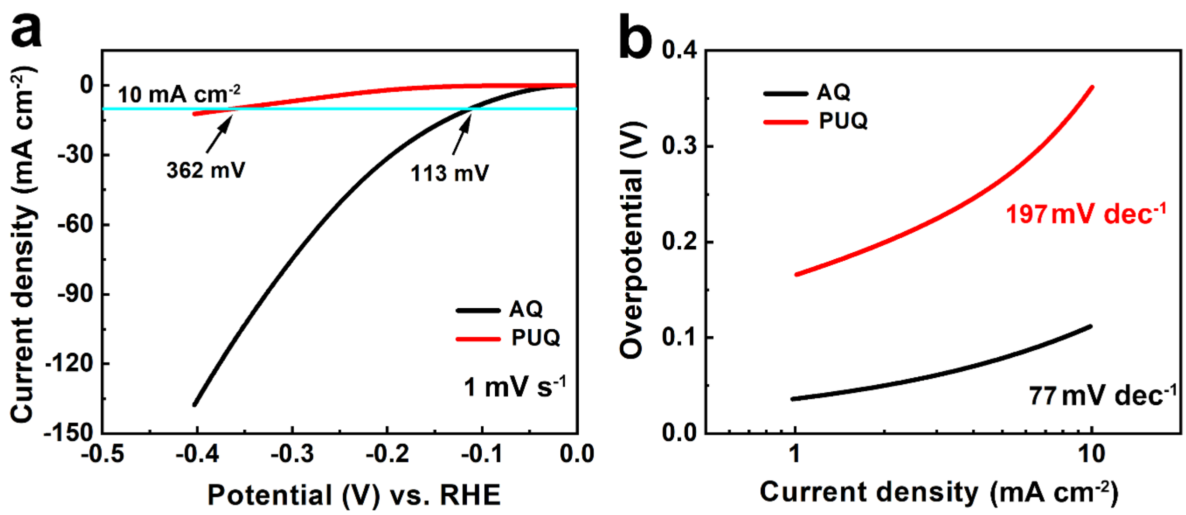

Figure S4. LSV polarization curves (a), Tafel plots (b) of the AQ and PUQ electrodes in 0.5 M H_2SO_4 solution

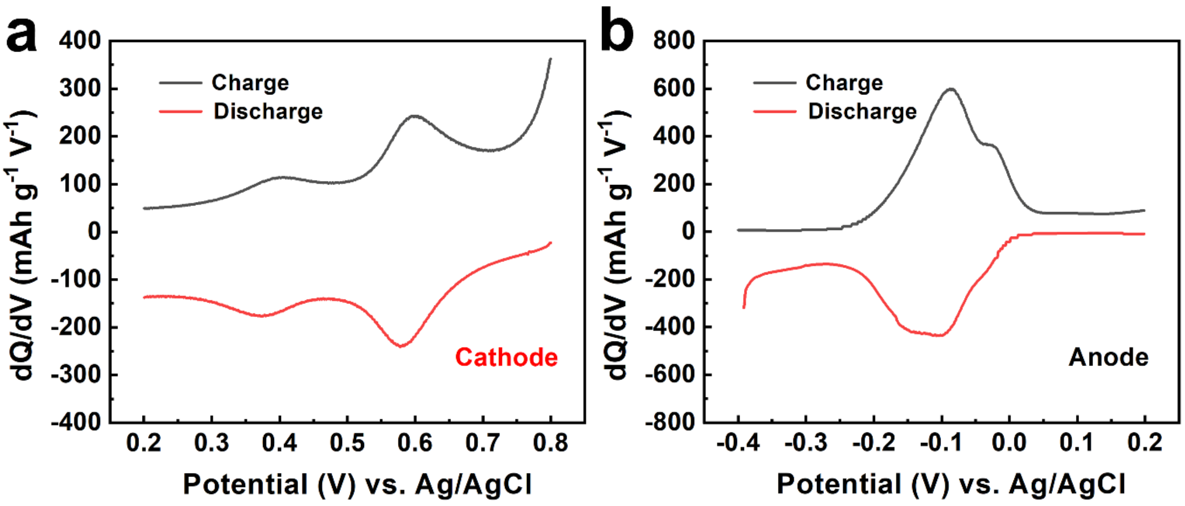

Figure S5. dQ/dV curves from GCD curves of the cathode (a) and anode (b).

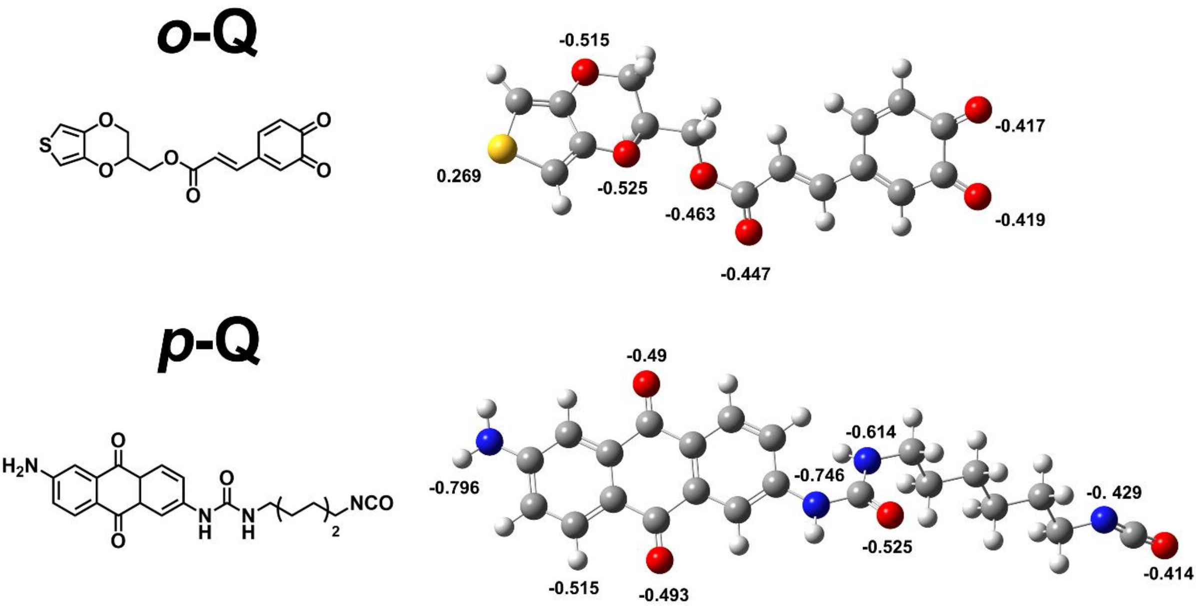

Figure S6. The charge distribution of oxygen, nitrogen, and sulfur atoms with *o*-Q and *p*-Q structures.

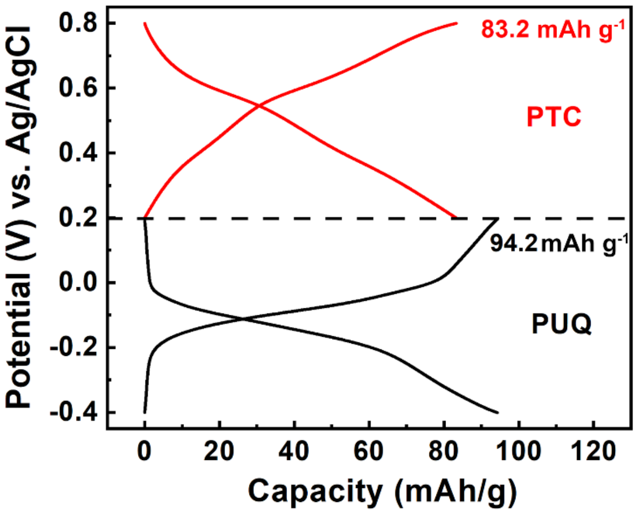

Figure S7. GCD curves of the cathode and anode at 1 A g^{-1}.

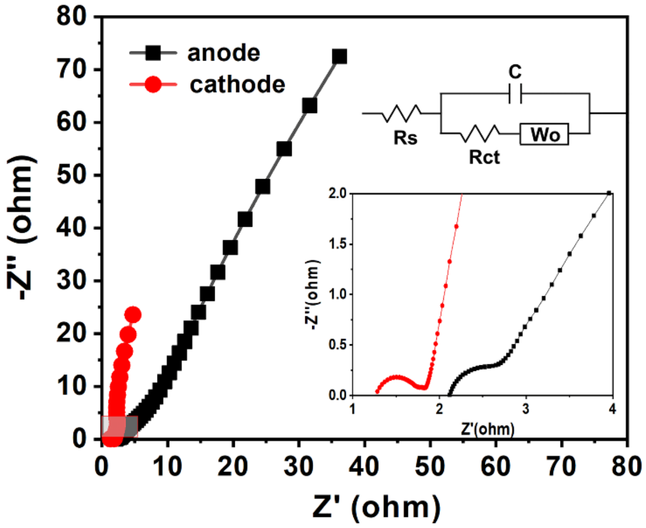

Figure S8. EIS of the anode and cathode ranges from 0.1 to 100k Hz (inset: equivalent circuit model).

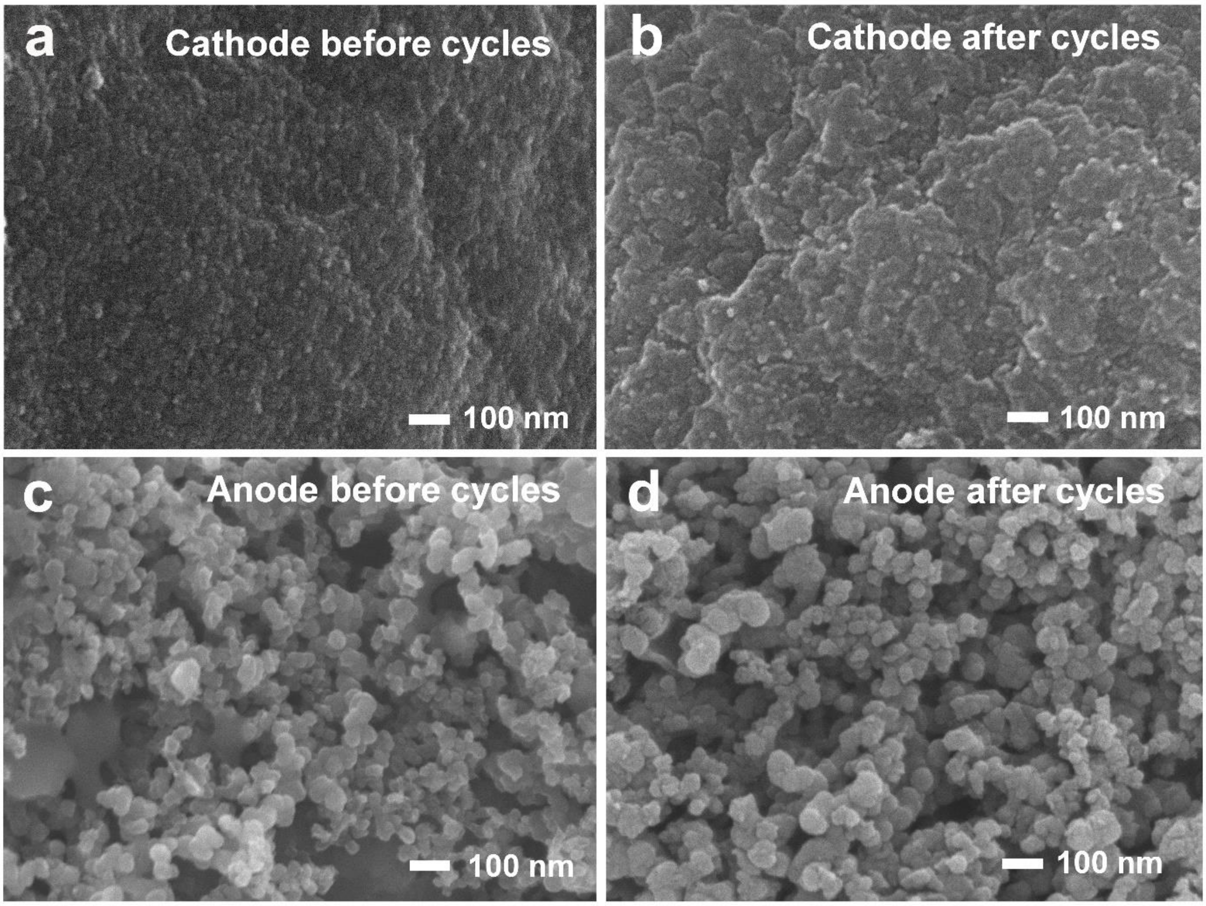

Figure S9. (a, c) SEM of the cathode and anode before cycles; (b, d) SEM of the cathode and anode after 1000 cycles at 2 A g^{-1}.
